# Phenotypic Characterization of Diffuse Large B-Cell Lymphoma Cells and Prognostic Impact

**DOI:** 10.3390/jcm8071074

**Published:** 2019-07-22

**Authors:** Julie Devin, Alboukadel Kassambara, Angélique Bruyer, Jérôme Moreaux, Caroline Bret

**Affiliations:** 1 CNRS UMR9002, Institute of Human Genetics, 34090 Montpellier, France; 2 Department of Biological Hematology, St Eloi Hospital, 34295 Montpellier, France; 3 University of Montpellier, Faculty of Medicine, 34090 Montpellier, France

**Keywords:** DLBCL, flow cytometry, biomarker, prognosis, drug resistance

## Abstract

Multiparameter flow cytometry (MFC) is a fast and cost-effective technique to evaluate the expression of many lymphoid markers in mature B-cell neoplasms, including diffuse large B cell lymphoma (DLBCL), which is the most frequent non-Hodgkin lymphoma. In this study, we first characterized by MFC the expression of 27 lymphoid markers in 16 DLBCL-derived cell lines to establish a robust algorithm for their authentication. Then, using the expression profile in DLBCL samples of the genes encoding B lymphoid markers that are routinely investigated by MFC, we built a gene expression-based risk score, based on the expression level of *BCL2, BCL6, CD11c*, and *LAIR1,* to predict the outcome of patients with DLBCL. This risk score allowed splitting patients in four risk groups, and was an independent predictor factor of overall survival when compared with the previously published prognostic factors. Lastly, to investigate the potential correlation between BCL2, BCL6, CD11c, and LAIR1 protein level and resistance to treatment, we investigated the response of the 16 DLBCL cell lines to cyclophosphamide, etoposide, doxorubicin, and gemcitabine. We found a correlation between BCL6 overexpression and resistance to etoposide. These results show the interest of MFC for the routine characterization of DLBCL cells and tumors samples for research and diagnostic/prognostic purposes.

## 1. Introduction

Diffuse large B-cell lymphoma (DLBCL) represents 30% to 40% of all non-Hodgkin lymphoma (NHL) cases in adults. It displays heterogeneous clinical and biological characteristics. Although most patients with DLBCL achieve long-term remission, a third of them relapse after standard treatment with the combination of rituximab, cyclophosphamide, doxorubicin, vincristine, and prednisone (R-CHOP) [1]. In the last decade, gene expression profiling of DLBCL cells allowed the identification of two main molecular subtypes that are related to the cell of origin and are associated with different clinical outcomes: germinal-center B-cell–like (GCB) and activated B-cell–like (ABC). The GCB subgroup (50% of all DLBCL) is associated with a good prognosis, and tumor cells display a healthy germinal-center B cell gene expression profile. The ABC subgroup (30% of cases) has a poorer outcome, and tumor cells display a healthy peripheral blood activated B cell gene expression profile, particularly an NF-*k*B signature. The remaining 20% of DLBCL correspond to primary mediastinal B cell lymphomas, or are unclassified [2,3]. This DLBCL subdivision is progressively incorporated in the clinical practice, due to the availability of methods to assess the cell of origin status and the development of novel therapeutic agents that target the different DLBCL subtypes [4]. In the clinic, DLBCL are usually classified using immunohistochemistry (IHC) methods and the Hans’ algorithm as well as molecular approaches, including the NanoString technology [5]. According to the Hans’ algorithm, DLBCL are subdivided in GCB tumors that are CD10+ or CD10-/BCL6+/MUM1-/IRF4-, and in non-GCB tumors that are CD10-/MUM1+/IRF4+ (BCL6 can be positive or negative) [6]. The Lymph2X assay is one of the molecular methods that use the NanoString technology and a 20-gene panel for cell of origin determination. It can also be used with formalin-fixed and paraffin-embedded tissue samples [7].

Multiparameter flow cytometry (MFC) is a fast and cost-effective technique that is widely used for the diagnosis and follow-up of lymphoproliferative disorders. In the clinical routine, it allows characterizing DLBCL cells in dissociated lymph node samples and also in blood, bone marrow, and body fluids, in the case of extra-nodal dissemination. In this study, we evaluated the interest of the routine DLBCL characterization by MFC using 16 DLBCL-derived cell lines and gene profiling data from cohorts of patients with DLBCL. First, we assessed whether MFC and a panel of 27 lymphoid markers could be used to develop a rapid method to authenticate DLBCL-derived cell lines for research purposes. Then, based on our previous experience in building powerful risk scores for multiple myeloma [8], acute myeloid leukemia [9], and B cell lymphoma [10], we developed a gene expression-based risk score for DLBCL at diagnosis using four independent cohorts of newly-diagnosed patients and the list of genes encoding the B lymphoid markers tested in our MFC-based routine DLBCL cell analysis. We found that this risk score, based on the expression of BCL2, BCL6, LAIR1/CD305, and CD11c, allows the identification of patients with high-risk DLBCL. Lastly, we analyzed the correlation between BCL2, BCL6, LAIR1/CD305, and CD11c protein level and the response to conventional treatments in the 16 DLBCL-derived cell lines.

## 2. Materials and Methods

### 2.1. Cell Culture

The 16 DLBCL cell lines (DOHH2, HT, OCI-LY19, DB, OCI-LY1, SUDHL-4, SUDHL-5, SUDHL-10, NUDHL-1, OCI-LY7, WSU-DLCL2, SUDHL-6, NUDUL-1, U2932, OCI-LY3, and RI-1) were purchased from the American Type Culture Collection or from DSMZ (Leibniz-Institut DSMZ-Deutsche Sammlung von Mikroorganismen und Zellkulturen GmbH, Germany). They were grown in RPMI-1640 Glutamax medium (Gibco, Invitrogen, Cergy Pontoise, France), supplemented with 10% fetal bovine serum (FBS) (PAA laboratory GmbH, Pasching, Austria) (U2932, SUDHL-4, HT, DOHH2, SUDHL-10, RI-1, and WSU-DLCL2 cells), 20% FBS (OCI-LY3, DB, SUDHL-5, NUDHL-1, and SUDHL-6 cells), or 15% FBS (NUDUL-1 cells). OCI-LY1, and OCI-LY7 cells were cultured in IMDM Glutamax (Gibco, Invitrogen, Cergy Pontoise, France), supplemented with 20% FBS, and OCI-LY19 cells in MEM alpha modified Glutamax (Gibco, Invitrogen, Cergy Pontoise, France) with 20% FBS. Cultures were maintained at 37 °C in a humidified atmosphere with 5% CO_2_. Contamination by *Mycoplasma* species was regularly monitored.

### 2.2. Flow Cytometry Analysis

Expression of 27 normal and pathological B lymphoid markers (CD19, CD20, FMC7, CD22, CD23, Kappa, Lambda, CD10, CD5, CD38, CD27, CD39, CD43, CD62L, CD81, CD200, BCL2, BCL6, Ki67, IgM, LAIR1, CD123, CD11c, CD25, CD103, CD71, and CD180) was evaluated by labeling the 16 DLBCL cell lines with specific antibodies. Surface staining of the cell suspension was performed at the recommended volume per test in the dark at room temperature (see supplementary Table S1 for references and combinations). Among the markers that were studied, BCL2, BCL6, and Ki67 were evaluated by intra-cytoplasmic staining, using the fix and perm solutions of the kit GAS-002 (Nordic-Mubio©, Susteren, The Netherlands).

Flow cytometry data were acquired with a Canto II flow cytometer (Becton Dickinson©, Le-Pont-de-Claix, France). The instrument setup and calibration were in accordance with the EuroFlow standard operating procedures [11,12].

For each marker, the mean intensity of fluorescence was evaluated after the initial gating of the cells of interest based on the CD45/SSC (side scatter) plot to exclude cell debris. Singlets were included in the FSC-H/FSC-A (forward scatter) plot, and events were analyzed, according to the EuroFlow panel guidelines. With the standard antibody panel, DLBCL-derived cell lines with a B-cell phenotype were selected based on the CD19/CD3 plot (or CD20/CD3 plot, if CD19 was weakly expressed). The total B-cell population represented the “OR” Boolean gate between the kappa-positive or lambda-positive B cell population. Events present on the kappa/lambda diagonal were removed [13]. For the other lymphoid markers, the total B-cell population gated on CD19 (or CD20) represented the “AND” Boolean gate.

### 2.3. Cell Viability Assay

DLBCL cells were cultured in 96-well flat-bottom microtiter plates in the appropriate medium with FBS in the presence of increasing concentrations of mafosfamide (Santa-Cruz Biotechnology, Dallas, TX, USA), doxorubicin or etoposide (Selleckchem, Houston, TX, USA), or the BCL6 inhibitor 79-6 (Calbiochem, San Diego, CA, USA) for 4 days.

The number of viable cells was determined using the Cell Titer Glo Luminescent Cell Viability Assay from Promega (Promega Corp, Madison, WI, USA). This test is based on the quantification of cellular ATP, as a marker of metabolically active cells, using a Centro LB 960 luminometer (Berthold Technologies, Bad Wildbad, Germany). Data were expressed as the mean percentage of six replicates and were normalized to the untreated control.

### 2.4. Gene Expression Profiling and Statistical Analyses

Gene expression microarray data were from four independent cohorts of patients with DLBCL. The first cohort (i.e., training cohort) included 233 patients treated with R-CHOP (R-CHOP Lenz cohort). Results were validated using the CHOP Lenz cohort (*n* = 181 patients treated with CHOP) [14], the Melnick cohort (*n* = 69 patients) [15], and the FFPE R-CHOP cohort (*n* = 72 patients) [7]. The patients’ pre-treatment clinical characteristics were previously described in References [7,14,15]. Affymetrix gene expression data are publicly available via the online Gene Expression Omnibus (http://www.ncbi.nlm.nih.gov/geo/) under the accession numbers GSE10846 (Lenz cohorts), GSE23501 (Melnick cohort) and GSE53786 (FFPE R-CHOP cohort) (Affymetrix HG-U133 plus 2.0 microarrays). Data were normalized with a Microarray Suite version 5.0 (MAS 5.0) using Affymetrix default analysis settings, and global scaling as the normalization method. The trimmed mean target intensity of each array was arbitrarily set to 500. Expression profiling data (accession number GSE56315) of DLBCL samples (*n* = 73) and normal centrocyte and centro-blast samples (*n* = 5/each) from human tonsils [16] were also compared.

One or several probe sets were available for 24 of the 27 B-cell markers evaluated by MFC. In the presence of several probe sets for the same gene, the probe set with the highest coefficient of variation was retained (Supplementary Table S1). Overall survival (OS) differences between groups were calculated using the log-rank test. Multivariate analysis was performed using the Cox proportional hazards model and Genomicscape (http://genomicscape.com) [17]. Survival curves were plotted using the Kaplan-Meier method. All analyses were done with R.2.10.1 and Bioconductor, version 2.5.

### 2.5. Building the B-Cell Marker Risk Score

The prognostic significance of probe sets was evaluated in the training cohort using the Maxstat R function, which allows us to determine the optimal cut point for continuous variables [18,19], and Benjamini Hochberg multiple testing correction, and then was validated in the validation cohorts [18,19].

Multivariate COX analysis was used to identify genes including the independent prognostic marker (i.e., *BCL6, BCL2, CD11c,* and *LAIR1*). Then, patients with DLBCL were split in five groups, according to their *BCL6, CD11c, BCL2,* and *LAIR1* expression profile [20]. Kaplan Meier analysis of these five different groups was performed and when two consecutive groups did not show any significant difference, they were merged.

### 2.6. Interaction Effect Quantification

For each combination, the percentage of expected growing cells in the case of effect independence was calculated, according to the Bliss equation [21,22].

*fuc* = *fuAfuB* (1)

where *fuc* is the expected fraction of cells unaffected by the drug combination in the case of effect independence, and *fuA* and *fuB* are the fractions of cells unaffected by treatment A and B, respectively. The difference between the fraction of living cells in the cytotoxicity test and the *fuc* was considered as an estimation of the interaction effect, with negative values indicating synergism and positive values antagonism.

## 3. Results

### 3.1. A Barcode to Identify DLBCL-Derived Cell Lines by MFC

**Figure 1:**
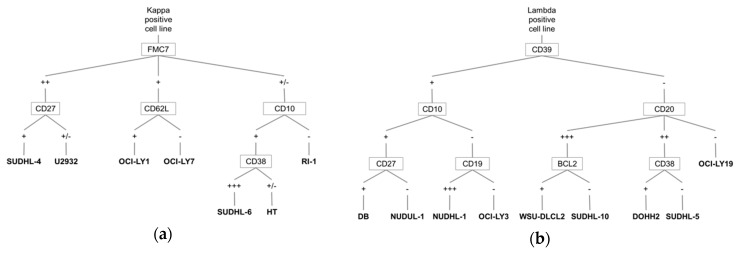
Algorithm for the identification of DLBCL derived-cell lines using multi-parameter flow cytometry. Diagram showing the identification strategy based on the mutually exclusive expression of the kappa (**a**) or lambda (**b**) light chain of immunoglobulins.

In our laboratory, we routinely use 16 DLBCL-derived cell lines that display a GCB profile (n = 12; DOHH2, HT, OCI-LY19, DB, OCI-LY1, SUDHL-4, SUDHL-5, SUDHL-10, NUDHL-1, OCI-LY7, WSU-DLCL2, and SUDHL-6) or an ABC profile (*n* = 4, NUDUL-1, U2932, OCI-LY3, and RI-1). To precisely determine their B-cell phenotype and check their identity using flow cytometry after each thawing, we evaluated the expression of 27 markers routinely investigated in mature B cell lymphoma (membrane markers: Kappa, Lambda, CD19, CD20, CD22, CD23, CD5, CD10, CD27, CD38, CD39, CD43, CD62L, CD81, CD200, FMC7, IgM, LAIR1, CD123, CD103, CD25, CD11c, CD71, and CD180, and intra-cytoplasmic markers: Ki67, BCL2, and BCL6) (Supplementary Table S2). We then generated an algorithm that is based on the mutually exclusive expression of the immunoglobulin kappa and lambda light chains, and on the selective expression (absent/present or low/bright expression) of specific markers within these two subgroups (Figure 1).

A first discrimination was done based on light chain expression (seven cell lines kappa-positive *versus* nine lambda-positive cell lines. In the kappa-positive cell lines (Figure 1A), the lymphoid marker FMC7 allowed discriminating three groups: bright, low, and near absent. Within the FMC7^bright^ group, CD27 expression separated SUDHL-4 (CD27^bright^) and U2932 (CD27^low^) cells. Within the FMC7^low^ group, CD62L discriminated between OCI-LY1 (CD62L^positive^) and OCI-LY7 (CD62L^negative^) cells, and, within the FMC7^negative^ group, CD10 was not expressed by RI-1. Among the CD10^positive^ cell lines, CD38 was bright in SUDHL-6 cells and weak in HT cells. In the kappa-positive group (Figure 1A), the lymphoid markers FMC7, CD27, CD62L, CD10, and CD38 allowed for discriminating the different cell lines.

In the lambda-positive group (Figure 1B), the different cell lines were separated using the lymphoid markers CD39, CD10, CD20, CD27, CD19, BCL2, and CD38.

We could separate the nine lambda-positive cell lines in two groups based on CD39 expression (Figure 1B). In the CD39^positive^ group, CD10 staining allowed us to form two other sub-groups (CD10^positive^ and CD10^negative^ cells). Among the CD10^positive^ cells, CD27 discriminated between NUDUL-1 (CD27^positive^) and DB (CD27^negative^) cells. Among the CD10^negative^ cells, CD19 allowed us to separate the NUDHL-1 (CD19^positive^) and OCI-LY3 (CD19^negative^) cell lines. In CD39^negative^ cells, the lymphoid marker CD20 identified three sub-groups: CD20^bright^, CD20^low^, and CD20^negative^ (OCI-LY19 cells). In the CD20^bright^ sub-group, BCL2 intracytoplasmic staining discriminated between WSU-DLCL2 (BCL2^positive^) and SUDHL-10 (BCL2^negative^) cells. Lastly, in the CD20^low^ sub-group, DOHH2 (CD38^positive^) and SUDHL-5 (CD38^negative^) were separated by CD38 staining.

We validated these results by performing a principal component analysis (PCA) incorporating MFI of the B-cell markers in our algorithm (Supplementary Figure S1). Validation of the algorithm with an independent set of DLBCL cell lines will be of interest.

### 3.2. CD39 is A Useful Marker to Discriminate Between ABC and GCB DLBCL Tumor Samples

**Figure 2:**
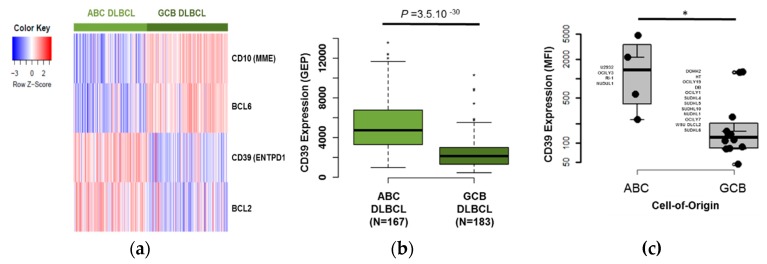
**CD39 expression discriminates between GCB and ABC DLBCL primary tumors and cell lines.** Cluster gram showing the expression of *MME, BCL6, ENTPD1*, and *BCL2* genes in ABC DLBCL samples (*n* = 167) and GCB DLBCL samples (*n* = 183) in the ABC and GCB DLBCL samples from the R-CHOP and CHOP Lenz cohorts (gene expression profile, GEP, data) (**a**). (B) Boxplots illustrate the expression signal (GEP) of *ENPD1* in ABC DLBCL samples and in GCB DLBCL samples in the Lenz cohort (**b**). Boxplots showing CD39 mean fluorescence intensity (MFI, logarithmic scale) in 16 DLBCL derived-cell lines (**c**). The boxes indicate the 25th and 75th percentile values. The line in the middle corresponds to the median. The vertical lines, to the 10th and the 90th percentiles. The outliers are identified as the third quartile plus 1.5 IQR (interquartile range); * *p*-value < 0.05 (Mann-Whitney U-test). GCB: germinal center B cell–like, ABC: Activated B cell–like, DLBCL: diffuse large B cell lymphoma.

We then performed a significance analysis of microarrays (SAM) analysis of the probe sets that correspond to the 27 markers that we routinely use for the characterization of pathological mature B cells by MFC to compare their expression in ABC and GCB DLBCL samples with the aim to find additional markers to categorize tumor samples. For this analysis, we used the gene expression profiling data of ABC and GCB DLBCL samples (*n* = 167 and *n* = 183, respectively) from the CHOP and R-CHOP Lenz cohorts (non-classified tumor samples were excluded). Four of these 27 genes were differentially expressed in these two groups. *CD10* and *BCL6* were upregulated in GCB samples, whereas *CD39* and *BCL2* were upregulated in ABC samples (Figure 2A). These results are in agreement with previous studies showing that *CD10, BCL6*, and *BCL2* are differentially expressed between ABC and GCB DLBCL [23] (*CD10* and *BCL6* are used in the Hans’ algorithm) and that *BCL2* is overexpressed in the ABC subtype [24]. Furthermore, we confirmed CD39 gene upregulation in ABC DLBCL samples (Figure 2B, *p* < 0.0001 ABC vs. GCB group) and also in the ABC DLBCL-derived cell lines compared with the GCB DLBCL-derived cell lines (*p* < 0.05) (Figure 2C).

### 3.3. The B Cell Marker Risk Score

**Figure 3:**
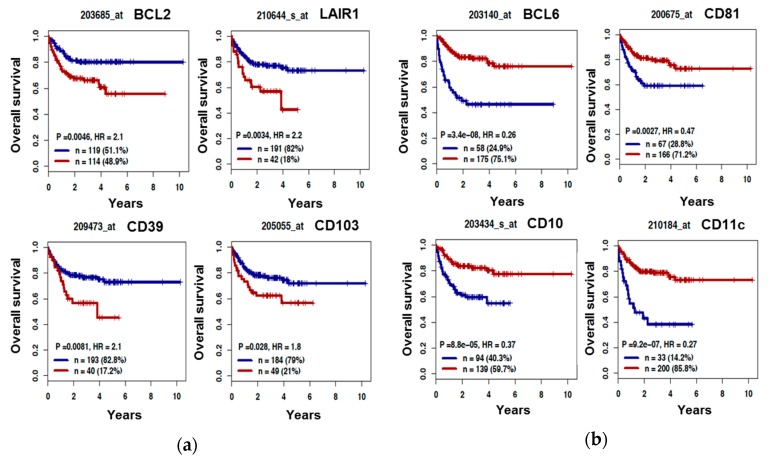
Eight lymphoid markers could predict the outcome of patients with DLBCL. Kaplan-Meier curves indicated that, in the training cohort (R-CHOP Lenz cohort, *n* = 233), high expression of *BCL2*, *LAIR1*, *CD39*, or *CD103* is associated with a poor outcome (i.e., overall survival in function of time) (**a**), whereas high expression of *BCL6*, *CD81*, *CD10*, or *Cd11c* is associated with better overall survival (**b**). Red, overexpression, and blue, downregulation. Curves were compared with the log rank test. HR: hazard ratio.

First, using the Maxstat R function [25], we investigated the prognostic value of the 27 B-cell markers in two cohorts of patients with DLBCL. High expression of *BCL2*, *LAIR1*, *CD39*, and *CD103* was associated with a poor outcome in both the R-CHOP Lenz cohort (training cohort, *n* = 233) and in the CHOP Lenz cohort (*n* = 181 patients, one of the validation cohorts) (Figure 3A and Supplementary Figure S2A). Conversely, high expression of *BCL6*, *CD10*, *CD11c*, and *CD81* was associated with a significant longer OS (Figure 3B and supplementary Figure S2B). We then investigated the expression of these eight markers in the ABC and GCB molecular subgroups (*n* = 167 and *n* = 183 samples, respectively, from the CHOP and R-CHOP Lenz cohorts, as above). *BCL2, CD39*, and *CD103* were significantly overexpressed in ABC DLBCL, whereas *BCL6, CD10, CD11c*, and *CD81* were overexpressed in GCB DLBCL (Supplementary Figure S3). These data were validated at the protein level only for CD39 (Figure 2C) and CD10 in the DLBCL-derived cell lines by MFC (Supplementary Figure S4).

**Figure 4:**
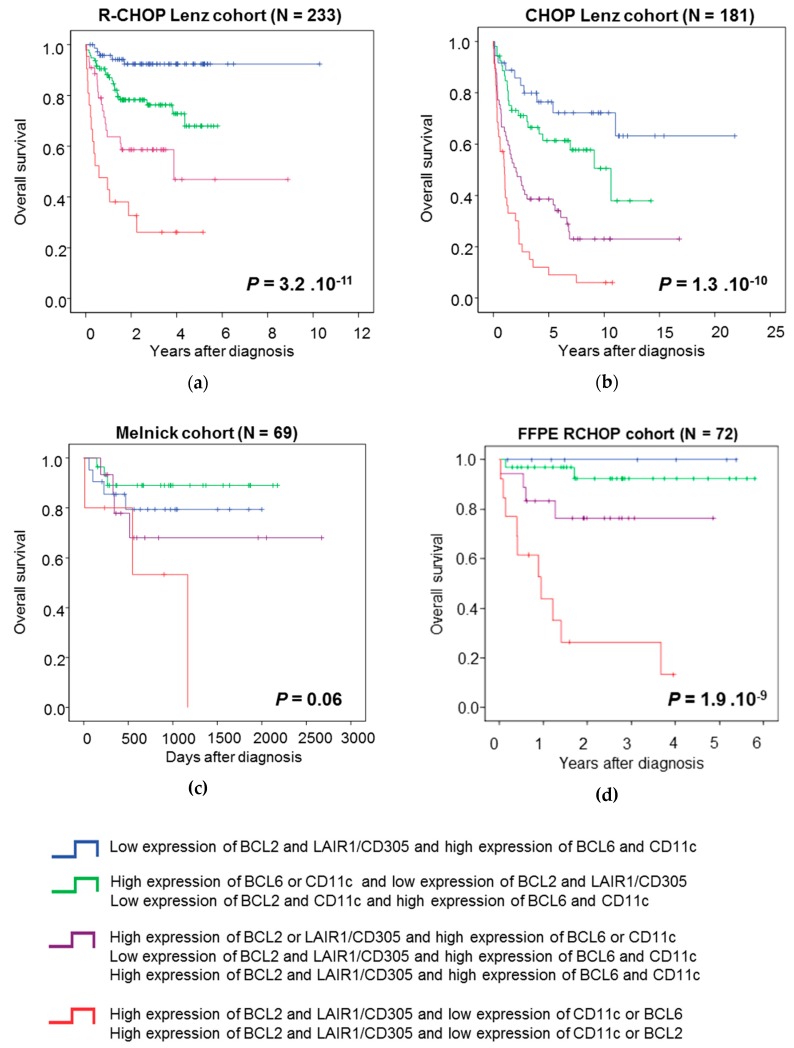
The risk score predicts the outcome of patients with DLBCL in four independent cohorts. Kaplan-Meier curves of overall survival in function of time in the R-CHOP Lenz cohort (**a**), CHOP Lenz cohort (**b**), R-CHOP Melnick cohort (**c**), and FFPE RCHOP cohort (**d**). Data were compared with the log rank test.

Using a multivariate COX analysis, we found that only *BCL6, BCL2, CD11c*, and *LAIR1* remained independent prognostic factors (Table 1). On the basis of these data, we used *BCL6, BCL2, CD11c*, and *LAIR1* gene expression to create a risk score. We split patients in the training cohort in five groups, according to the tumor *BCL6, CD11c, BCL2,* and *LAIR1* expression (high/low), and then performed Kaplan Meier analyses to determine the overall survival (OS) in the function of the expression level. This allowed us to identify four groups with significantly different OS (Figure 4A). Group 1 (very low risk, 31.3% of all patients) comprised patients with low *BCL2* and *LAIR1/CD305* expression and high *BCL6* and *CD11c* expression. Group 2 (low risk, 40.8% of patients) comprised patients with high *BCL6* or *CD11c* expression and low *BCL2* and *LAIR1/*CD305 expression, or low *BCL2* and *CD11c* expression and high *BCL6* and *CD11c* expression. Group 3 (medium risk, 18.9% of patients) comprised patients with high *BCL2* or *LAIR1/CD305* expression and high *BCL6* or *CD11c* expression, or low *BCL2* and *LAIR1*/CD305 expression and high *BCL6* and *CD11*c expression, or high *BCL2* and *LAIR1*/CD305 expression and high *BCL6* and *CD11c* expression. Group 4 (high risk, 9% of patients) included patients with high *BCL2* and LAIR1/CD305 expression and low CD11c and BCL6 expression, or high *BCL2* and *LAIR1*/CD305 expression and low *CD11c* or *BCL2* expression. Group 1 and group 2 did not reach the median OS, whereas group 3 and group 4 had a median OS of 46 months and 9 months, respectively (*p* = 3.2E-11, Figure 4A). The prognostic value of the score was validated in the CHOP Lenz cohort (*n* = 181) and in the FFPE R-CHOP cohort (*n* = 72), and showed a trend (*p* = 0.06) in the Melnick cohort (*n* = 69) (Figure 4B–D).

**Figure 5:**
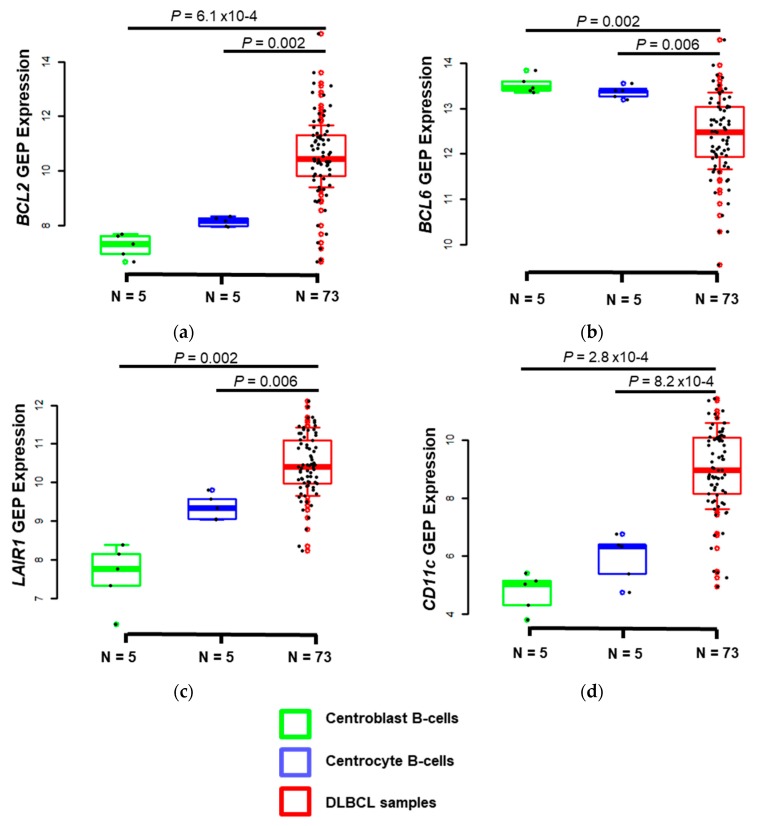
Comparison of *BCL2* (**a**), *BCL6* (**b**), *LAIR1* (**c**), and *CD11c* (**d**) gene expression in DLBCL samples and in normal centrocytes and centro-blasts (GSE12195 dataset). The box-plot diagrams include the median value and the interquartile rage (IQR). The error bars represent the minimum values under the median and the outliers are identified as the third quartile plus 1.5 IQR (R I386 3.4.0 software, R Foundation). Results were compared using the Mann-Whitney U-test.

We then asked whether the four genes included in the prognostic risk score (*BCL2*, *BCL6*, *LAIR1*, and *CD11c*) were differentially expressed between malignant and normal B cells. To this aim, we compared their expression in normal centrocytes (*n* = 5), normal centroblasts (*n* = 5), and DLBCL samples (*n* = 73) (GSE12195 dataset) [26]. *BCL2, LAIR1,* and *CD11c* were significantly overexpressed in DLBCL samples compared with normal centrocytes (*p* = 0.002, *p* = 0.006 and *p* = 0.00008, respectively) and centro-blasts (*p* = 0.0006, *p* = 0.002 and *p* = 0.0003, respectively) (Figure 5). Conversely, *BCL6* was downregulated in DLBCL compared with normal centrocytes (*p* = 0.006) and centro-blasts (*p* = 0.002) (Figure 5). Previous studies showed that the BCL2 transcript and protein expression are low in germinal center cells [27], and that BCL6 is strongly expressed in centro-blasts and centrocytes compared with memory B cells and plasma-blasts in a normal population of tonsil B cells [28].

Moreover, *CD10* and *CD103* were significantly downregulated in DLBCL samples compared with normal centrocytes (*p* = 0.0004 and *p* = 0.013, respectively) and centroblasts (*p* = 0.0002 and *p* = 0.004, respectively). *CD39* was significantly overexpressed in DLBCL samples compared with normal centrocytes (*p* = 0.0007) and centroblasts (*p* = 0.001). *CD81* expression was comparable in the three groups (Supplementary Figure S5).

These results suggest that BCL2, LAIR1, and CD11c and BCL6 could be used to discriminate DLBCL cells from normal B cells during MFC analysis of dissociated tumor samples.

We then compared, in the R-CHOP Lenz cohort (*n* = 233 patients), the prognostic value of our risk score and of other previously identified prognostic factors, including the ABC and GCB molecular subgroups, age, International prognostic Index (IPI), and DNA repair score (Table 1A). In two-by-two comparisons, the risk score, age, IPI, and DNA repair score remained independent prognostic factors of OS (Table 1B). When all prognostic factors were tested together in multivariate COX analyses, only the risk score and the DNA repair score maintained their prognostic value (Table 1C).

Moreover, univariate COX analysis showed that the individual lymphoid markers *BCL2, CD39, CD103, CD81, LAIR1, BCL6, CD5*, and *CD11c* had a prognostic value in the Lenz R-CHOP cohort (Table 2A). When we tested all parameters together, *BCL2, BCL6, CD5, LAIR1*, and *CD81* expression remained significant (Table 2B).

To verify the applicability of our risk score in routine flow cytometry analysis, we correlated the gene expression profile signal of the markers included in the risk score with their mean fluorescence intensity (MFI) in the 16 DLBCL cell lines, due to the absence of a high enough number of primary tumor cell samples for a statistically robust analysis, and found a significant correlation for all of them (r = 0.68 for BCL2, r = 0.59 for BCL6, r = 0.80 for LAIR1, and r = 0.84 for CD11c, *p* < 0.05, Supplementary Figure S6).

### 3.4. BCL6 Protein Expression is Correlated with DLBCL Cell Response to Etoposide

**Figure 6:**
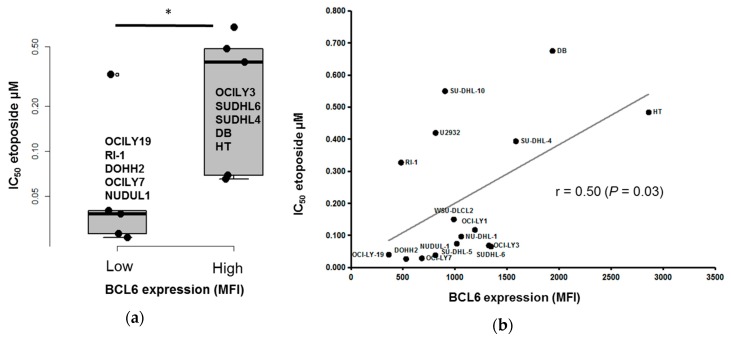
BCL6 expression correlates with the response to etoposide in DLBCL cell lines. Box-plots illustrate the inhibitory concentration 50 (IC_50_) of etoposide on a logarithmic scale. The boxes represent the 25th and 75th percentile values. The line in the middle corresponds to the median. The vertical lines correspond to the 10th and the 90th percentiles, and the circles, to the outliers. * *p* value < 0.05 (Mann-Whitney U-test) (**a**). Linear regression analysis of BCL6 expression (MFI) in function of the IC_50_ of etoposide in 16 DLBCL cell lines. r represents the Spearman correlation coefficient, *p* value <0.05 (Spearman correlation test) (**b**).

Lastly, we investigated the correlation between BCL2, LAIR1, BCL6, CD5, and CD11c expression in MCF and response to doxorubicin, mafosfamide, and etoposide in the 16 DLBCL cell lines. Only BCL6 expression was significantly and positively correlated with etoposide resistance (i.e., the half maximal inhibitory concentration, IC_50_) (r = 0.50, *p* < 0.05) (Figure 6). Since BCL6 is the most frequently involved oncogene in DLBCL, we then determined whether BCL6 inhibition could overcome etoposide drug resistance in DLBCL cell lines. To this aim, we used the low molecular weight compound 79-6 that binds to the corepressor binding groove of the BCL6 BTB domain. This BCL6 inhibitor inhibits tumor growth in mice xenografted with DLBCL cells without any toxicity [29]. A synergy combining 79-6 with low etoposide concentrations was identified (Supplementary Figure S7A). These data suggest that high BCL6 expression could be of interest to identify etoposide-resistant patients. BCL6 targeting could present a therapeutic interest to overcome etoposide resistance (Supplementary Figure S7A).

## 4. Discussion

DLBCL cell lines are widely used in laboratories to explore the cellular and molecular mechanisms involved in DLBCL pathogenesis. However, routine controls, such as cell authentication and contamination testing, are required to minimize behavioral changes due to cross-contamination with other cell lines, mycoplasma contamination, or phenotypic modifications caused by extensive culturing [30,31]. Therefore, a rapid and low-cost method for cell identity confirmation after each thawing is required, as currently done for mycoplasma detection [32]. A variety of methods are available for authentication testing. Among them, short tandem repeat profiling, which relies on a polymerase chain reaction-based assay to assess polymorphic tetra-nucleotide or penta-nucleotide repeats, has been recommended for human cell lines [30]. Nevertheless, this technique has some limitations, including possible genetic drift with continuous cell passaging [33]. In this case, we describe a simple phenotype-based method for the routine authentication of DLBCL cell lines using MFC. This test can be performed in about half day and does not require DNA extraction and analysis. Moreover, flow cytometry could be a useful method for the detection of very low cross-contamination, the risk of which can increase during extensive culture time and repeated freezing-thawing cycles. However, more attention should be provided to instrument and setting calibration for multiparameter MFC profiling. Validation of the algorithm using an independent set of DLBCL cell line will be important.

Our B-cell biomarkers could be used for different applications, such as discriminating ABC and GCB DLBCL, predicting the outcome of patients with DLBCL, and providing information related to the treatment response. For instance, we found that CD39 could be an additional marker to discriminate between ABC and GCB DLBCL tumors and cell lines, and that its upregulation in DLBCL is associated with a poor outcome (Figure 3). CD39 is encoded by the *ENTPD1* gene, and is a typical cell surface enzyme with a catalytic site that faces the extracellular compartment. CD39 is an ectonucleotidase that catalyzes the hydrolysis of γ- and β-phosphate residues of nucleoside tri-phosphates and di-phosphates into mono-phosphate derivatives [34]. Cardoso et al. showed that CD39 expression analysis by flow cytometry is useful to discriminate B-cell lymphomas, particularly Burkitt lymphoma and ABC DLBCL [35].

In the clinical practice, DLBCL samples are classified according to their putative cell of origin by IHC and in the different subgroups using the Hans’s algorithm. By comparing the classifications obtained by IHC and gene expression profiling, Gutierrez-García et al. showed that the Hans’s algorithm misclassified 48% and 15% of GCB and ABC tumors, respectively. CD39 staining could be of interest to help better define the cell of origin in patients with DLBCL [36].

In current practice, patients with DLBCL are classified according the Ann Arbor classification system. IPI, age-adjusted IPI, and cell of origin are associated with a prognostic value [7,37,38,39]. More recently, gene-based risk scores have been proposed [10,40]. In the present study, we assessed the prognostic value of genes that encode lymphoid markers and built a risk score based on the expression of *BCL2*, *BCL6*, *LAIR1*, and *CD11c*. Our risk score allowed dividing patients with DLBCL in four risk groups (low risk, intermediate risk, and high risk). This approach is an independent predictor factor for OS when compared with the previously published prognostic factors.

These data also suggest that deregulated expression of genes from different signaling pathways is involved in DLBCL pathophysiology. For instance, BCL2 and BCL6 are two markers linked to germinal center B cells [41]. BCL2 is an anti-apoptosis factor located in the mitochondrial membrane and is important in normal B-cell development and differentiation. In DLBCL, the association of *MYC* and *BCL2* rearrangements (double-hit lymphoma, DHL) or of *MYC* and *BCL2* overexpression (double expresser lymphoma, DEL) has a significant prognostic value. These tumors show a more aggressive behavior and more frequent treatment failure after conventional therapy than non-DHL/non-DEL tumors [42,43]. DHL is a B-cell lymphoma that carries rearrangements between *MYC* and another oncogene, usually *BCL2* and more rarely *BCL6, BCL3*, or *CCND1*. DHL and triple-hit lymphoma represent about 10% of all DLBCL [44]. The t(14;18)(q21;q32)/IGH-BCL2 translocation is observed in 20% to 30% of DLBCL, usually in the GCB group [45]. DEL, in which both *MYC* and *BCL2* are overexpressed, represent 25% to 35% of all DLBCL, and are more often classified in the ABC/non-GCB rather than in the GCB group. DEL are associated with poor prognosis compared with non-DEL that overexpress either *MYC* or *BCL2* [46,47]. BCL2 overexpression was associated with bad prognosis in our risk score. In the previously published BCL2 IHC score [48], patients with BCL2-overexpressing DLBCL benefitted from treatment with venetoclax. Venetoclax is a highly selective BCL2 inhibitor and is considered a breakthrough therapy for refractory or relapsed chronic lymphocytic leukemia [49]. It is currently in phase I/II development as monotherapy and/or combination therapy for NHL (including DLBCL, mantle cell lymphoma, and follicular lymphoma) and acute myeloid leukemia [50].

BCL6 is a DNA-binding protein and a transcriptional repressor involved in mediating growth suppression, and in blocking the terminal differentiation of B lymphocytes. *BCL6* has also been implicated in class switch recombination and somatic hypermutation [41]. In GCB-DLBCL, gain-of-function mutations of *EZH2* cooperate with *BCL6* overexpression to inhibit centro-blast terminal differentiation [51]. In ABC-DLBCL, constitutive activation of BCL6 by chromosomal translocations and loss of function of *PRDM1* block the terminal differentiation of B lymphocytes to plasma cells [52]. The most common chromosomic translocation in DLBCL (30% of cases) involves BCL6 on chromosome 3q27 and IGH on chromosome 14q32. However, BCL6 has many other potential translocation partners [45].

A recent meta-analysis found that, in the absence of a concomitant *MYC* translocation, the *BCL2* and *BCL6* translocation status does not provide any additional prognostic information for OS or progression free survival (PFS) [53].

Leukocyte-associated immunoglobulin-like receptor-1 (LAIR1), which is also known as CD305, is a member of the immunoglobulin (Ig) superfamily, and is a type І transmembrane glycoprotein. LAIR-1 includes a C2-type Ig-like domain and two immuno-receptor tyrosine-based inhibition motifs (ITIMs). It is broadly expressed by nearly all immune cells, such as T-cells, B-cells, and natural killer (NK) cells. The known LAIR1 ligands are extracellular matrix collagen and C1q, which is the first complement component. In vivo, LAIR1 inhibits B-cell receptor (BCR)-mediated signaling and controls kinase pathways involved in B-cell proliferation [54,55,56]. LAIR1 overexpression was associated with a bad prognosis in pediatric acute lymphoblastic leukemia (ALL) [57]. Unlike normal pre-B cells, patient-derived ALL cells express *LAIR1* at high levels. Genetic studies revealed that LAIR1 critically modulates oncogenic signaling through recruitment of the inhibitory phosphatases PTPN6 and INPP5D. High LAIR1 expression blocks hyperactivation of the BCR pathway by inhibition of SYK, SRC, and the ERK kinase that promotes negative selection of auto-reactive B cells during B-cell development [58]. LAIR1 is also a biomarker of the “host response” cluster in the DLBCL classification described by Morin and colleagues [59]. The presence of tumor-infiltrating lymphocytes and increased expression of multiple components of the T-cell receptor (TCR) complex (TCR alpha and beta and CD3 subunits), CD2, and additional molecules associated with T/NK-cell activation and the complement cascade characterized the “host response” cluster [59]. The “host response” cluster in DLBCL displays an inflammatory/immune response and also high expression of monocyte/macrophage and dendritic cell markers. LAIR1 expression may be related to the tumor environment inflammatory response.

CD11c is an integrin alpha X chain protein (*ITGAX* gene). Integrins are heterodimeric integral membrane proteins composed of an alpha chain and a beta chain. CD11c combines with the beta 2 chain (*ITGB2*) to form a leukocyte-specific integrin, referred to as inactivated-C3b (iC3b) receptor 4 (CR4). CD11c is expressed in dendritic cells, macrophages, and monocytes and is a differentiation marker of inflammation. Moreover, CD11c is a diagnostic marker of hairy cell leukemia, and *ITGAX* expression is associated with aggressive prostate cancer [60]. The prognostic value of CD11c varies depending on the tumor type. For instance, CD11c expression in chronic lymphocytic leukemia cells is associated with a significantly higher incidence of Richter’s transformation [61]. Conversely, in gastric cancer, high CD11c expression is associated with a decreased risk of death and relapse, and may be regarded as an alternative indicator of a favorable prognosis [62]. CD11c is also a marker of dendritic cells. It has been reported that CD11c-positive dendritic cells promote the formation of germinal centers in the steady state and their response after immunization [63]. Furthermore, it has been suggested that, in follicular lymphoma, the presence of CD11c-positive dendritic cells in the tumor micro-environment is involved in tumor progression or recurrence by supporting regulatory T-cell infiltration [64].

Since GEP is not included in the current routine diagnostic work-up, immunohistochemistry or flow cytometry represent interesting substitutes. The validation of our risk score at the protein level using flow cytometry or immunohistochemistry could lead to a simple, fast, and cheaper prognostic tool for routine DLBCL diagnosis and prognosis. Interestingly, BCL2, BCL6, LAIR1, and CD11c expression by flow cytometry has already been reported in studies of normal and malignant B cells including clinical significance analyses [11,65,66,67,68]. Flow cytometry or immunohistochemistry analyses will allow us to analyze, simultaneously, the microenvironment that is recognized as being indirectly a reflection of the tumor clone and presenting a major impact in clinical course and response to treatment. Furthermore, it will be important to better understand the functional role of BCL2, BCL6, LAIR1, and CD11c in DLBCL pathogenesis and pathophysiology.

We found that high BCL6 expression, determined by flow cytometry, was associated with significantly decreased etoposide-induced toxicity. Kurosu et al. showed that, in B cell lymphoma, BCL6 overexpression inhibits the increase in ROS levels and apoptosis in response to etoposide and other chemotherapeutic reagents [69]. Moreover, we observed that the BCL6 inhibitor 79-6 could synergize with low concentrations of etoposide in DLBCL-derived cell lines. A recent study reported that BCL6 depletion induces BCL2 and BCL-XL upregulation in DLBCL cells. According to this observation, in most DLBCL cell lines, effective tumor cell killing requires the concomitant inhibition of BCL6 and BCL2 [70]. We validated the synergy between concomitant inhibition of BCL2 and BCL6 (Supplementary Figure S7B). Furthermore, combination of etoposide with IC_20_ of 79-6 and IC_20_ of Venetoclax demonstrated a significantly higher toxicity on DLBCL cells (Supplementary Figure S7D). These findings suggest that the combination of BCL2 and BCL6 inhibitors could be of interest to potentiate etoposide-induced toxicity in BCL6-overexpressing DLBCL cells.

## 5. Conclusions

We developed a flow cytometry-based algorithm for the authentication of DLBCL-derived cell lines used in research to limit the risk of cross-contamination or misidentification. Moreover, our risk score allows the identification of patients with high-risk DLBCL. Since it is based on the expression of four genes that encode markers routinely analyzed by MFC or IHC, it could represent a powerful tool for simple outcome prediction in DLBCL.

## Figures and Tables

**Table 1:** Cox univariate and multivariate analyses of prognostic factors of overall survival in patients with diffuse large B-cell lymphoma (R-CHOP Lenz cohort, *n* = 233).

**A.**	**Overall Survival (*n* = 233)**	
**Prognostic Variable**	**HR**	***p*-Value**
Age (>60 years)	2.20	<0.0001
GCB-ABC molecular subgroups	2.75	<0.0001
IPI	1.79	<0.0001
DNA repair score	3.87	<0.0001
Risk Score	2.41	<0.0001
**B.**	**Overall Survival (*n* = 233)**	
**Prognostic Variable**	**HR**	***p*-Value**
Risk Score	2.248	<0.0001
Age (>60 years)	1.846	0.03
Risk Score	2.269	<0.0001
GCB-ABC molecular subgroups	1.359	NS
Risk Score	2.113	<0.0001
IPI	1.582	<0.0001
Risk Score	2.263	<0.0001
DNA repair score	2.909	<0.0001
**C.**	**Overall Survival (*n* = 233)**	
**Prognostic Variable**	**HR**	***p*-Value**
Age (>60 years)	1.80	NS
GCB-ABC molecular subgroups	2.23	NS
IPI	0.20	NS
DNA repair score	3.44	<0.0001
Risk Score	1.61	0.007

The indicated prognostic factors were tested individually (A), two by two (B), or in multivariate analysis (all variables) (C) using a Cox regression model. *p*-values and hazard ratios (HR) are shown. NS: not significant at the 5% threshold. The IPI groups were defined as follows: low risk group = IPI score 0 or 1, low-intermediate risk group = IPI score 2, high-intermediate risk group = IPI score 3, and high-risk group = IPI score 4 or 5. IPI, international prognostic index. GCB, germinal-center B-cell–like subgroup. ABC, activated B cell–like subtype.

**Table 2:** Cox univariate and multivariate analyses of the effect of lymphoid markers on overall survival in patients with diffuse large B-cell lymphoma (gene expression data from the R-CHOP Lenz cohort, *n* = 233).

**A.**	**Overall Survival (*n* = 233)**	
**Lymphoid Marker**	**HR**	***p-*Value**
BCL2	2.11	0.006
BCL6	0.26	<0.0001
CD39	2.1	0.01
CD103	1.82	0.03
CD11c	0.27	<0.0001
LAIR1	2.24	0.004
CD10	0.37	<0.0001
CD5	0.59	0.04
**B.**	**Overall Survival (*n* = 233)**	
**Lymphoid Marker**	**HR**	***p-*Value**
BCL2	1.96	0.01
BCL6	0.44	0.007
CD39	1.22	NS
CD103	1.30	NS
CD11c	0.33	<0.0001
LAIR1	2.82	0.002
CD10	0.39	NS
CD5	0.47	0.01

The indicated prognostic factors were tested as single variables (A) or multi-variables (B) using a Cox-regression model. *p*-values and hazard ratios (HR) are shown. NS: not significant at the 5% threshold.

## Supplementary Materials

The following are available online at https://www.mdpi.com/2077-0383/8/7/1074/s1, Figure S1: Principal component analysis representing MFI of FCM biomarkers used for DLBCL cell lines discrimination in our identification algorithm. Figure S2: Survival curves of patients with DLBCL in the CHOP Lenz cohort divided in two groups according to the expression of the prognostic lymphoid markers. Figure S3: Expression of prognostic markers in the ABC and GCB DLBCL samples from the Lenz cohorts (*n* = 350). Figure S4: CD10, BCL2, BCL6, CD103, LAIR1, CD81 and CD11c expression in GCB and ABC DLBCL-derived cell lines. Figure S5: Comparison of CD10, CD81, CD39 and CD103 gene expression in DLBCL samples and normal centrocytes and centroblasts (GSE12195 dataset). Figure S6: Correlation between gene expression of the prognostic lymphoid markers (Affymetrix microarrays) and their protein expression in 16 DLBCL cell lines by MFC. Figure S7: Effect of the combination of etoposide (increasing concentrations) and the BCL6 inhibitor 79-6 in DLBCL-derived cell lines. Table S1: Antibodies references. Table S2: Mean fluorescence intensity of 27 lymphoid markers in 16 DLBCL-derived cell lines obtained by multiparameter flow cytometry.

## Abbreviations

DLBCL	Diffuse large B-cell lymphoma
GEP	Gene expression profiling
COO	Cell of origin
GCB	Germinal-center B-cell–like subgroup
ABC	Activated B cell–like subtype
R	Rituximab
CHOP	Cyclophosphamide, doxorubicin, vincristine, and prednisone
EFS	Event-free survival
OS	Overall survival
FCM	Flow cytometry
FSC	Forward scatter
SSC	Side scatter
NF-*k*B	Nuclear factor *k*B
ERK	Extracellular signal-regulated kinases
IC_50_	Inhibitory concentration 50
INPP5D	Src homology 2 domain containing inositol polyphosphate 5-phosphatase 1
IHC	Immunohistochemistry
PCR	Polymerase chain reaction
PTPN6	Protein Tyrosine Phosphatase Non-Receptor Type 6
SAM	Significance analysis of microarray
STR	Short tandem repeat
SYK	Spleen tyrosine kinase
