# Supplementary material for: Phenotypic Characterization of Diffuse Large B-Cell Lymphoma Cells and Prognostic Impact
Source: J Clin Med. 2019 Jul 22;8(7):1074. doi: 10.3390/jcm8071074 (PMC6678649; doi:10.3390/jcm8071074)
Supplement: Supplementary file 1 [file jcm-08-01074-s001.pdf]

Journal of
Clinical Medicine

Supplementary Table S1. Antibodies References.

Panel 1: (surface staining only)

Antibody	Brand	Reference	Fluorochrome
CD8	BD©	555366	FITC
Kappa	Agilent©/Dako©	F0434	FITC
Lambda	Agilent©/Dako©	R04371-2	PE
CD56	BD©	345812	PE
CD5	BD©	341109	PerCP Cy5.5
CD19	BD©	341113	PE Cy7
CD10	BD©	332777	APC
CD20	BD©	655872	V450
CD3	BD©	641415	APC H7
CD4	BD©	560345	V450
CD45	BD©	560777	V500

Panel 2 (surface staining only):

Antibody	Brand	Reference	Fluorochrome
FMC7	BD©	332786	FITC
CD23	BD©	332782	PE
CD5	BD©	341109	PerCP Cy5.5
CD19	BD©	341113	PE Cy7
CD81	BD©	551112	APC
CD38	BD©	560676	Alexa Fluor 700
CD43	BD©	655407	APC H7
CD22	BD©	563940	BV421
CD45 (HI30)	BD©	560777	V500
CD200	BD©	562853	BV605
CD3	BD©	560365	V450
Kappa	Agilent©/Dako©	F0434	FITC
Lambda	Agilent©/Dako©	R043701-2	PE

Panel 3 (surface staining only):

Antibody	Brand	Reference	Fluorochrome
CD103	BD©	333155	FITC
LAIR1	BD©	550811	PE
CD123	BD©	560826	PE Cy7
Kappa	BD©	341108	APC
CD11c	BD©	561352	Alexa Fluor 700
CD25	BD©	562660	BV605
CD19	BD©	641395	APC H7
Lambda	BD©	562893	BV421
CD45 (HI30)	BD©	560777	V500
CD3	BD©	332771	PerCP Cy5.5
CD22	Beckman Coulter©	IM3704	PerCP Cy5.5

Panel 4:

Antibody	Brand	Reference	Fluorochrome
		Surface staining:	
CD5	BD©	341109	PerCP Cy5.5
CD19	BD©	341113	PE Cy7
Kappa	BD©	561319	Alexa Fluor 700
CD3	BD©	641415	APC H7
Lambda	BD©	561379	V450
CD45 (HI30)	BD©	560777	V500
CD10	BD©	562978	BV605
		Intracytoplasmic staining:	
Bcl2	BD©	563600	Alexa Fluor 647
Ki67	Agilent©/Dako©	F726801	FITC
Bcl6	BD©	561522	PE

Panel 5: (Surface staining only)

Antibody	Brand	Reference	Fluorochrome
CD5	BD©	341109	PerCP Cy5.5
CD19	BD©	341113	PE Cy7
Kappa	BD©	561319	Alexa Fluor 700
CD3	BD©	641415	APC H7
Lambda	BD©	561379	V450
CD45 (HI30)	BD©	560777	V500
CD10	BD©	562978	BV605
CD62L	BD©	347443	FITC
CD39	BD©	555464	PE
CD27	BD©	337169	APC

Panel 6 (surface staining only)

Antibody	Brand	Reference	Fluorochrome
CD180	BD©	551953	PE
IgM	Agilent©/Dako©	F0058	FITC
CD5	BD©	341109	PerCP Cy5.5
CD19	BD©	341113	PE Cy7
CD3	BD©	641415	APC H7
CD10	BD©	562978	BV605

Supplementary Table S2. Mean fluorescence intensity of 27 lymphoid markers in 16 DLBCL-derived cell lines obtained by multiparameter flow cytometry. GCB: germinal center B cell–like, ABC: Activated B cell–like.

**Cell line**	**DOHH2**	**HT**	**OCILY19**	**DB**	**OCILY1**	**SUDHL4**	**SUDHL5**	**SUDHL10**	**NUDHL1**	**OCILY7**	**WSU DLCL2**	**SUDHL6**	**U2932**	**OCILY3**	**RI-1**	**NUDUL1**
**Marker**	GCB	GCB	GCB	GCB	GCB	GCB	GCB	GCB	GCB	GCB	GCB	GCB	ABC	ABC	ABC	ABC
**Kappa**	0	242	0	0	21,929	80,047	0	0	0	49,655	0	867	14,883	0	50,300	0
**Lambda**	23,589	0	7888	115,161	0	0	65,391	56,152	69,320	0	17,539	0	0	22,345	0	42,423
**CD5**	84	156	85	475	199	333	939	0	583	184	105	271	636	203	1230	238
**CD10**	20,342	19,936	5296	12,887	33,438	17,600	5876	4666	500	8513	11,571	35,062	3588	361	182	5266
**CD19**	45,038	30,247	33,935	1644	18,227	35,553	27,081	23,097	109,784	18,769	34,756	67,363	13,364	786	2870	65,938
**CD20**	48,832	2659	893	85,391	82,330	231,200	48,601	215,051	44,410	67,830	193,210	38,272	133,411	20,689	15,584	98,607
**CD22**	4314	4578	1366	3844	33,539	13,825	8349	15,267	11,331	4884	24,761	13,928	7251	6814	4807	3389
**CD23**	169	158	111	488	345	39	159	834	5765	530	342	384	762	1282	110	5487
**CD27**	3998	3650	317	12,969	3181	50,491	1815	27,880	289	2012	13,661	4204	1143	355	1973	735
**CD38**	8731	9277	10,053	1246	8497	13,578	0	5139	1208	31,492	6202	59,338	15,262	533	18,635	48,891
**CD39**	130	46	80	1240	152	86	81	112	1272	108	135	253	231	4682	573	2144
**CD43**	8833	8235	11,302	3309	18,439	8964	5032	14,796	6768	27,047	12,081	46,276	21,423	1580	18,825	64,381
**CD62L**	2438	1885	1122	4072	9361	311	607	1628	446	535	466	3154	1082	28,927	237	830
**CD81**	108,831	50,997	19,734	92,374	96,123	157,052	140,550	222,183	19,459	87,379	199,980	83,016	135,829	7732	68,932	200,134
**CD200**	981	578	206	1260	682	611	424	630	3721	688	1019	641	377	1078	369	380
**FMC7**	2815	270	253	3713	8044	55260	12,416	33,624	2056	3329	21,638	900	27,391	1161	277	7789
**ki67**	8433	21,152	5757	7350	5293	13,359	8762	8355	21,600	5269	5013	6881	4978	6739	5480	8509
**BCL2**	1229	301	2130	8250	7968	2780	421	491	7418	561	5198	2809	11,743	3243	8667	1656
**BCL6**	530	2862	363	1937	1191	1585	1017	904	1059	681	988	1345	813	1325	481	811
**IgM**	0	0	88	183	1817	271	2043	196	2148	1089	0	884	900	166	629	84
**LAIR1**	238	184	11,117	321	589	319	169	316	22,250	134	382	493	348	305	148	6712
**CD123**	245	187	441	135	587	142	124	213	488	170	384	250	340	611	419	635
**CD11C**	24	0	62	0	0	0	0	0	166	0	0	3	0	136	0	0
**CD25**	486	83	214	1412	480	261	957	944	453	1926	245	329	335	1341	287	981
**CD103**	228	67	249	213	264	314	207	139	2958	97	182	626	440	359	173	354
**CD71**	11,012	21,393	2892	4847	6179	6105	9028	15,479	1729	2592	4226	3287	15,939	38,175	1620	2712
**CD180**	811	14,359	106	1020	6463	3862	969	3757	793	1102	2772	1898	1490	223	1233	1359

Supplementary figures:

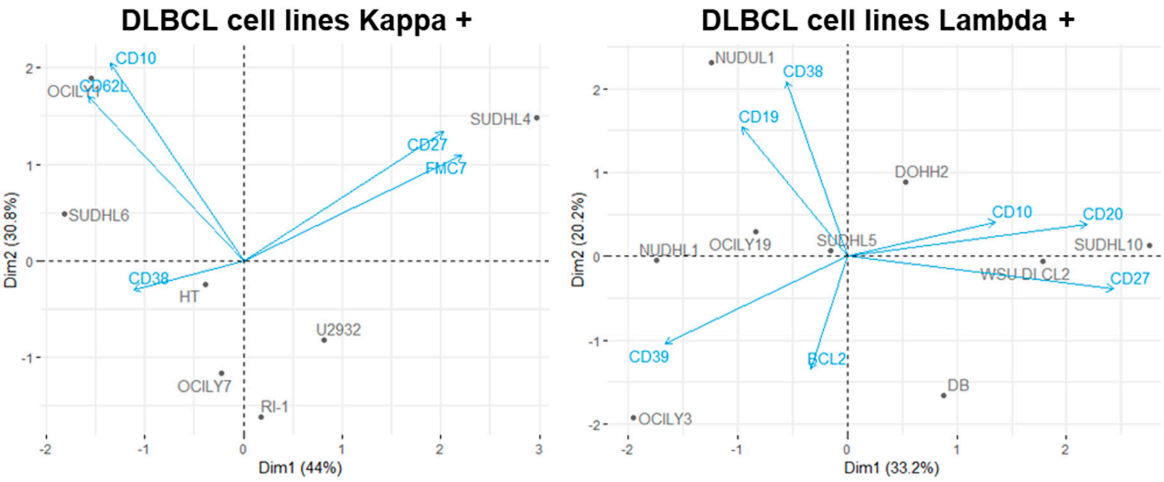

(**a**) (**b**)

Supplementary Figure S1. Principal component analysis representing MFI of FCM biomarkers used for DLBCL cell lines discrimination in our identification algorithm. The arrows represent the correlation of the variables and the distance between the variables and the origin measure the quality of variables representation. The points represent the coordinates of the individuals (cell lines). Kappa cell lines are presented on the panel (**a**) and lambda cell lines on the panel (**b**).

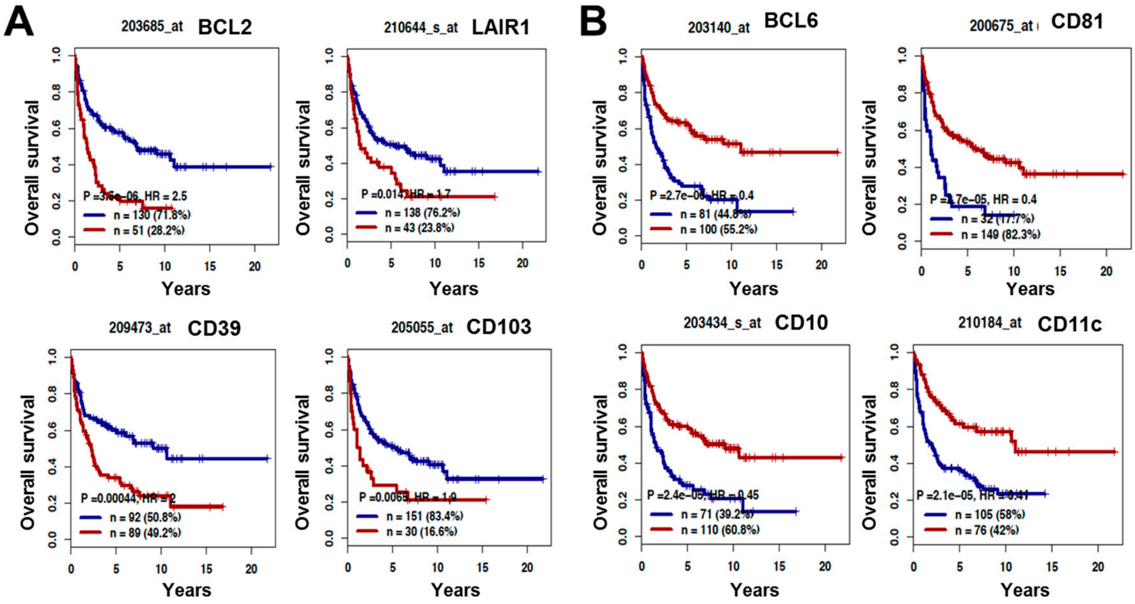

Supplementary Figure S2. Survival curves of patients with DLBCL in the CHOP Lenz cohort divided in two groups, according to the expression of the prognostic lymphoid markers. Kaplan-Meier curves show that, in the CHOP Lenz cohort (n = 181, validation cohort), high expression of *BCL2*, *LAIR1*, *CD39*, or *CD103* is associated with poor outcome (i.e., overall survival in function of time) (**A**), whereas high expression of *BCL6*, *CD81*, *CD10*, or *Cd11c* is associated with better overall survival (**B**). Red, overexpression, and blue, downregulation. Curves were compared with the log rank test.

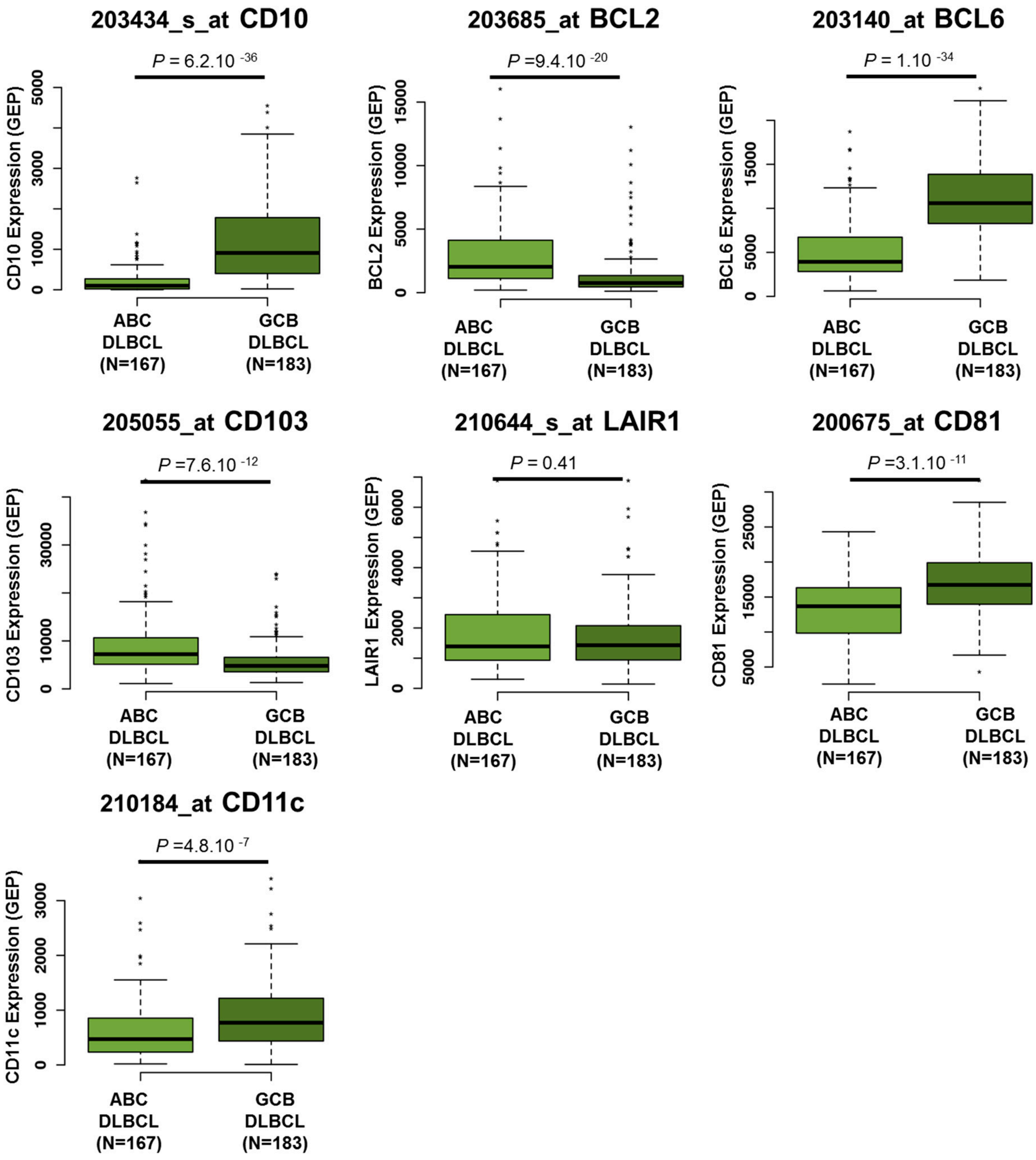

Supplementary Figure S3. Expression of prognostic markers in the ABC and GCB DLBCL samples from the Lenz cohorts (n = 350). Box-plots illustrate the expression of CD10, BCL2, BCL6, CD103, LAIR1, CD81, and CD11c (from the gene expression profile dataset of the Lenz cohorts). Boxes represent the 25th and 75th percentile values, the line in the middle corresponds to the median, the vertical lines indicate the 10th and the 90th percentiles, and the circles show the outliers. P values were calculated with the Mann-Whitney U-test. GCB: germinal center B cell–like diffuse large B cell lymphoma, ABC: Activated B cell–like diffuse large B cell lymphoma.

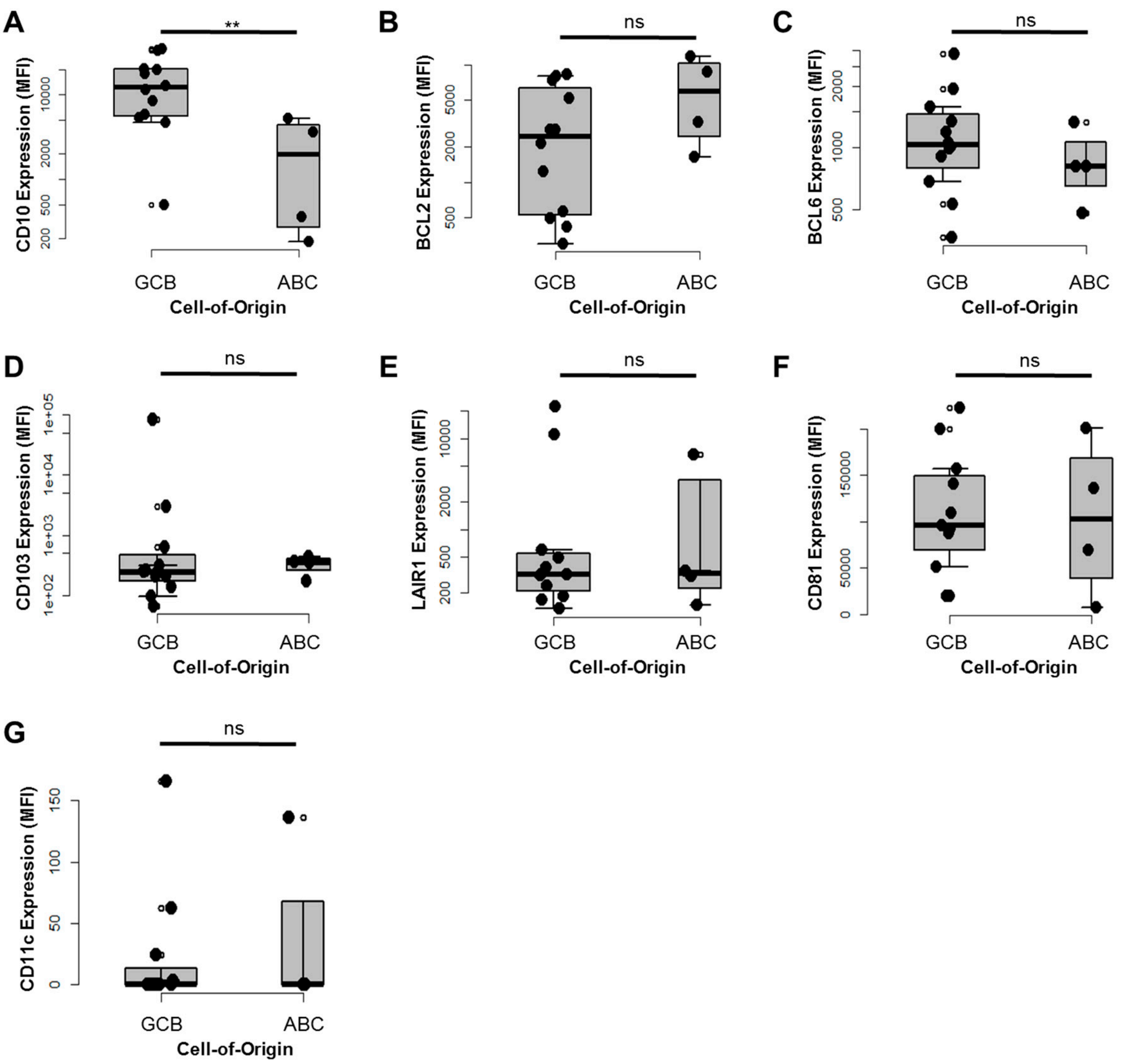

Supplementary Figure S4. CD10, BCL2, BCL6, CD103, LAIR1, CD81, and CD11c expression in GCB and ABC DLBCL-derived cell lines. Box-plots illustrate the mean fluorescence intensity (MFI) of CD10 (**A**), BCL2 (**B**), BCL6 (**C**) CD103 (**D**), LAIR1 (**E**), CD81 (**F**), and CD11c (**G**). Boxes represent the 25th and 75th percentile values, the line in the middle corresponds to the median, the vertical lines indicate the 10th and the 90th percentiles, and the circles show the outliers. ** p value < 0.01, ns: non-significant (Mann-Whitney U-test). GCB: germinal center B cell–like diffuse large B cell lymphoma, ABC: Activated B cell–like diffuse large B cell lymphoma.

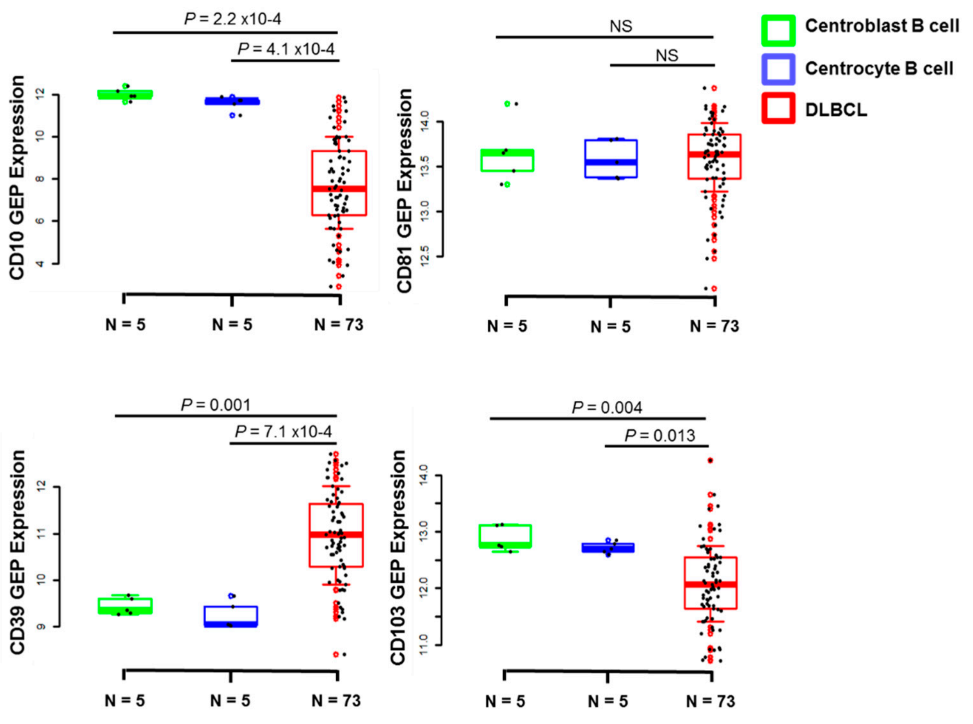

Supplementary Figure S5. Comparison of *CD10*, *CD81*, *CD39*, and *CD103* gene expression in DLBCL samples and normal centrocytes and centro-blasts (GSE12195 dataset). The box-plot diagrams show the median value and the interquartile rage (IQR). The error bars represent the minimum value under the median, and the outliers are identified as the third quartile plus 1.5 IQR (R I386 3.4.0 software). Results were compared using the Mann-Whitney U-test.

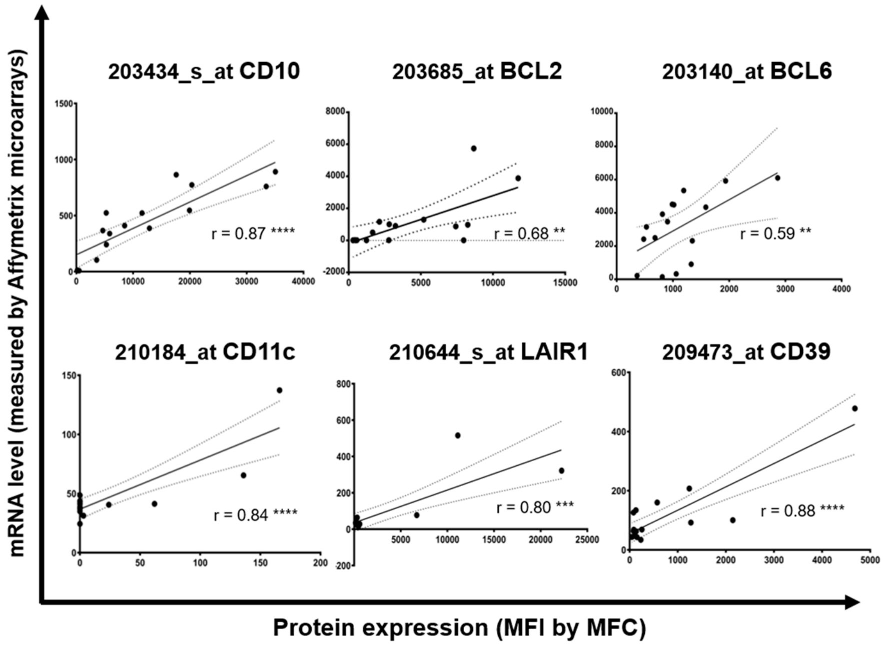

Supplementary Figure S6. Correlation between gene expression of the prognostic lymphoid markers (Affymetrix microarrays) and their protein expression in 16 DLBCL cell lines by MFC. Linear regression analysis of CD10, BCL2, BCL6, CD11c, LAIR1, and CD39 protein expression (MFI) in the 16 DLBCL-derived cell lines versus their mRNA level in DLBCL tumors (measured by Affymetrix microarrays). r represent the Pearson correlation coefficient, $n = 16$, ** $p < 0.01$, *** $p < 0.001$, **** $p < 0.0001$.

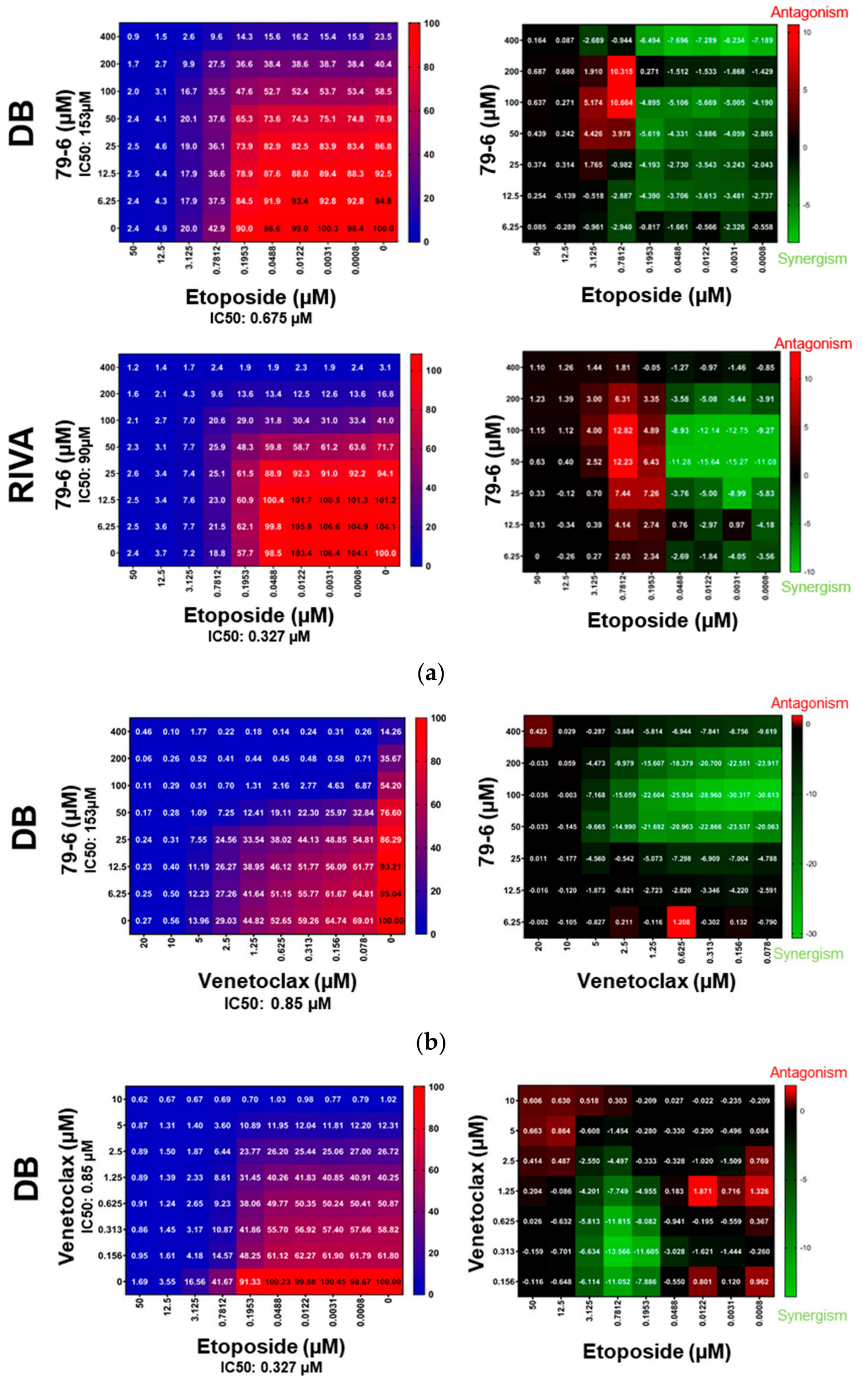
DB
79-6 (μM)
IC50: 153μM
Etoposide (μM)
IC50: 0.675 μM
Antagonism
Synergism
Etoposide (μM)
79-6 (μM)
RIVA
79-6 (μM)
IC50: 90μM
Etoposide (μM)
IC50: 0.327 μM
Antagonism
Synergism
Etoposide (μM)
79-6 (μM)
(a)
DB
79-6 (μM)
IC50: 153μM
Venetoclax (μM)
IC50: 0.85 μM
Antagonism
Synergism
Venetoclax (μM)
79-6 (μM)
(b)
DB
Venetoclax (μM)
IC50: 0.85 μM
Etoposide (μM)
IC50: 0.327 μM
Antagonism
Synergism
Etoposide (μM)
Venetoclax (μM)
(c)

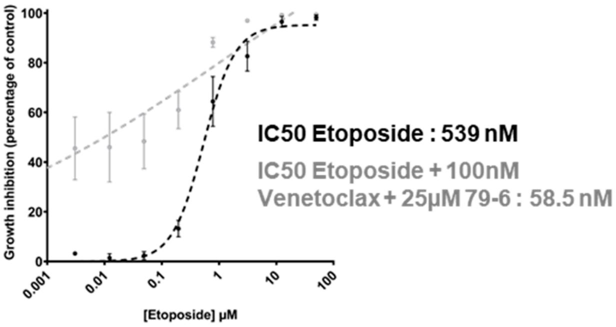

(**d**)

Supplementary Figure S7. Effect of the combination of etoposide (increasing concentrations) and the BCL6 inhibitor 79-6 in DLBCL-derived cell lines. The indicated DLBCL-derived cell lines were treated with increasing concentrations of etoposide combined with 79–6 (**a**), Venetoclax combined with 79-6 (**b**), or etoposide combined with Venetoclax (**c**) for 96 hours and cell viability was tested by ATP quantification to obtain the viability matrix. The synergy matrix was calculated as described in Materials and Methods. The DB DLBCL cell line was incubated with increasing concentrations of etoposide with IC_{20} of 79-6 (25μM) and IC_{20} of Venetoclax (100nM) for 96 hours. (**d**). Data are expressed as the mean percentage of three experiments and then normalized to the untreated control.
